# Supplementary figures and images for: Simulated climate change, but not predation risk, accelerates Aedes aegypti emergence in a microcosm experiment in western Amazonia
Source: PLoS One. 2020 Oct 20;15(10):e0241070. doi: 10.1371/journal.pone.0241070 (PMC7575111; doi:10.1371/journal.pone.0241070)

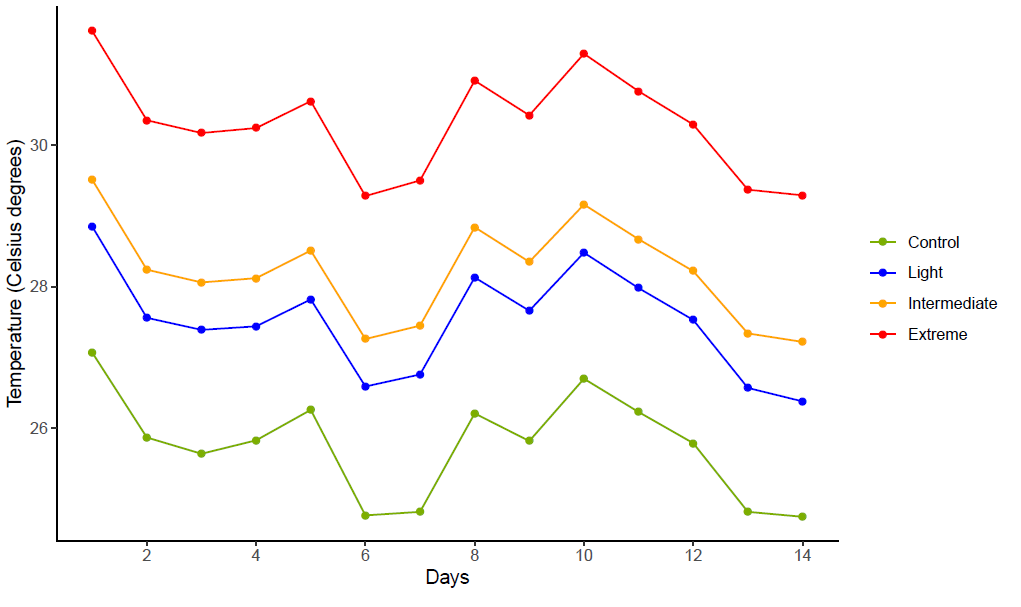

Supplement: S1 Fig — (TIF) [file pone.0241070.s001.tif]

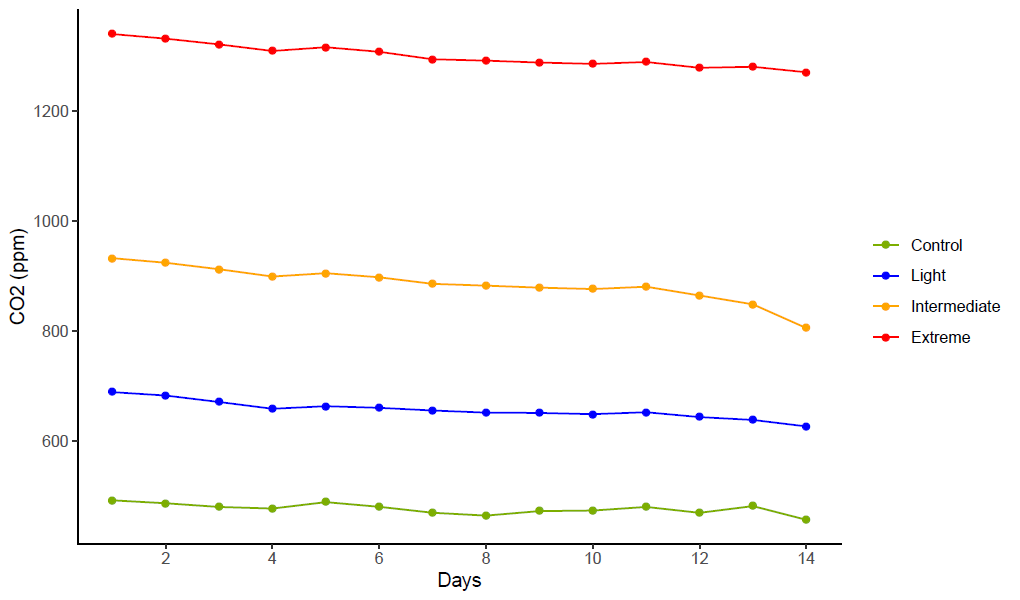

Supplement: S2 Fig — (TIF) [file pone.0241070.s002.tif]

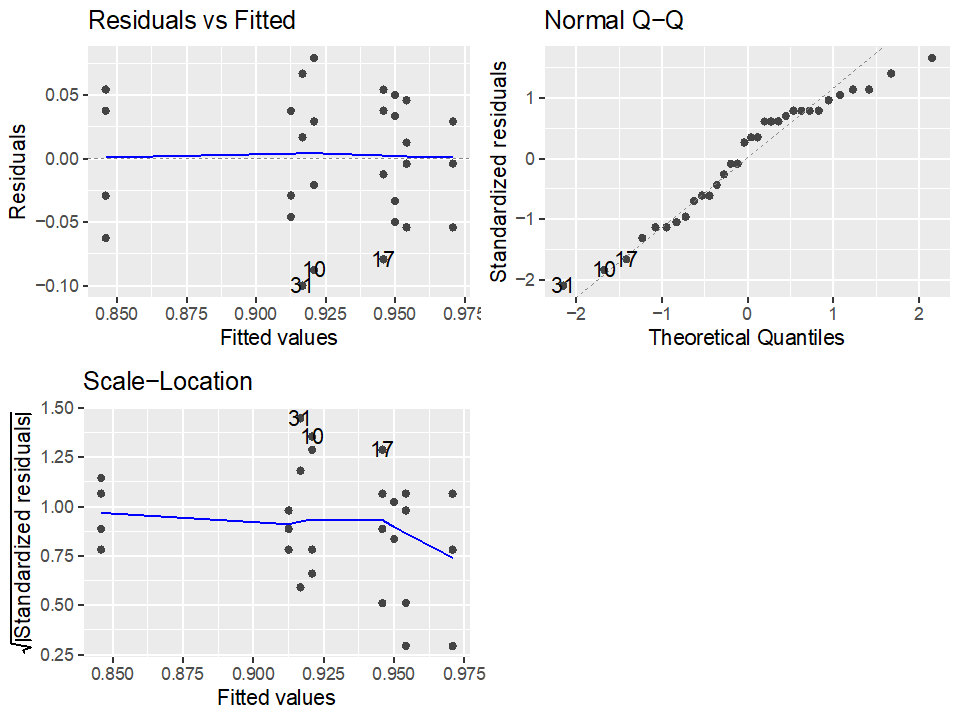

Supplement: S3 Fig — (TIF) [file pone.0241070.s003.tif]

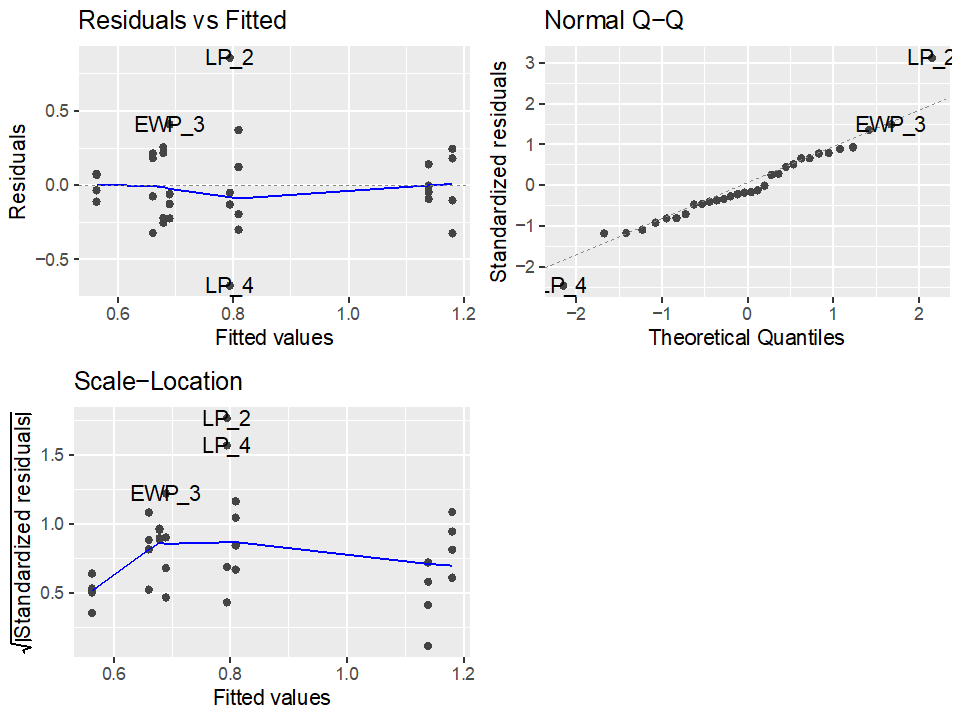

Supplement: S4 Fig — (TIF) [file pone.0241070.s004.tif]

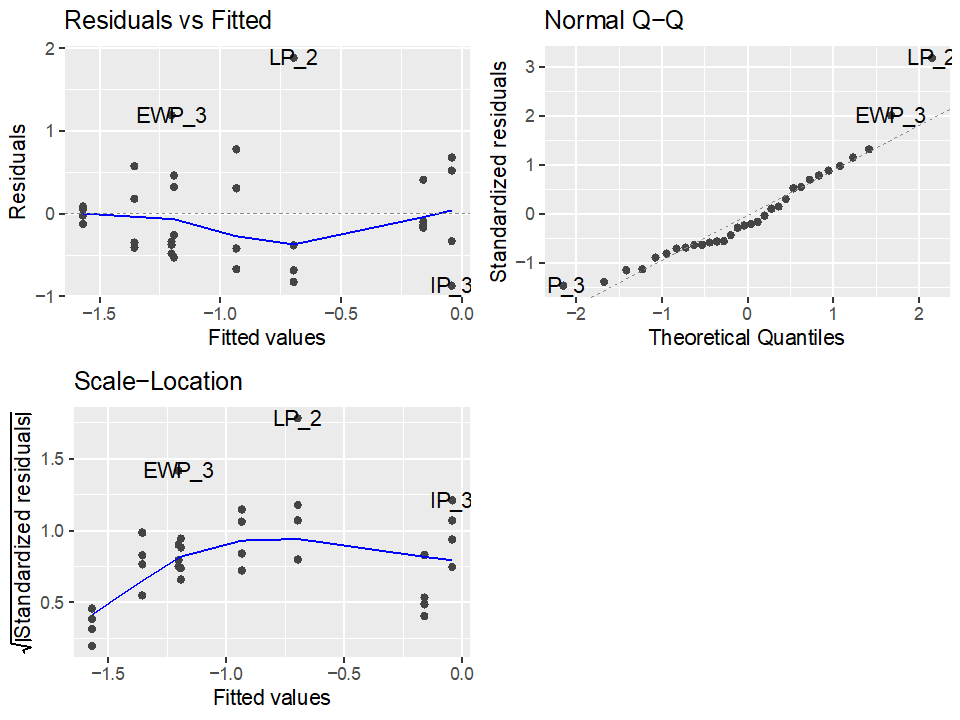

Supplement: S5 Fig — (TIF) [file pone.0241070.s005.tif]
