# Supplementary material for: Simulated climate change, but not predation risk, accelerates Aedes aegypti emergence in a microcosm experiment in western Amazonia
Source: PLoS One. 2020 Oct 20;15(10):e0241070. doi: 10.1371/journal.pone.0241070 (PMC7575111; doi:10.1371/journal.pone.0241070)
Supplement: Attachment — Submitted filename: BorgesReview.docx [file pone.0241070.s007.docx]

In this study, the authors simulated the impacts of future temperature and CO2 conditions as well as predator presence on adult emergence and larval survivorship. They found no impact of the simulated climate conditions or predation risk on larval survivorship, but did find that adult emergence pattern was affected by the simulated climate conditions, with earlier, more uniform emergence under the intermediate climate conditions.

Overall comment:

Abstract

Line 30: I would remove “those concerning” for clarity.

Introduction

Line 64: I would change to Around the world, some 390 million people are infected with dengue virus each year

Line 108: Remove mainly

Methods

Line 215-219: This could be made a little clearer if you explicitly state what your response variable is for the linear mixed model. I think it is number of emergences per day, but the reader might not be 100% certain.

Line 223-234: I think this is a really interesting way to analyze your data and you explained everything very thoroughly.

Results

Line 249: Could you specify how much higher the emergence rate was in the Light vs Control SCCS?

Discussion

Line 279-280: Since you only actually saw the accelerated development time under the intermediate climate change scenario, I would specify that here.

Line 285: Since your larvae weren’t under actual threat of predation (just subjected to chemical cues of predators), I don’t know that you have the grounds to broadly state that climatic variables are a stronger ecological driver than predation risk.

Line 308: I would substitute predation risk with predation cues here.

Line 326-327: Should just say “emergence distribution of *Ae. aegypti.*” without including adults.

Line 328-329: Since you just saw the effects in the Intermediate treatment, I would be interested to see a discussion of others that have seen a similar non-effect in the Extreme scenario where conditions are too hot and no longer speeding up development.

Line 338: Change to “It is important to carry out…”

Line 345: Same as previous comments, you can’t really say *Ae. aegypti* develops faster under SCCS—just the larvae develop faster under the *Intermediate* SCCS
