# Supplementary material for: Simulated climate change, but not predation risk, accelerates Aedes aegypti emergence in a microcosm experiment in western Amazonia
Source: PLoS One. 2020 Oct 20;15(10):e0241070. doi: 10.1371/journal.pone.0241070 (PMC7575111; doi:10.1371/journal.pone.0241070)
Supplement: Attachment — Submitted filename: Response to Reviewers.docx [file pone.0241070.s008.docx]

Dear Dr. Jiang-Shiou Hwang,

Academic Editor of PLOS ONE

PONE-D-20-07577R1

Simulated climate change, but not predation risk, accelerates *Aedes aegypti* emergence in a microcosm experiment in western Amazonia

We are grateful for the valuable suggestions and comments of the reviewers to our manuscript. As requested, we revised the manuscript incorporating the edits suggested by the reviewers. Our point-by-point answers to reviewers comments can be found below. The suggested changes were made directly into the text, and are marked in track changes.

Reviewer 1:

**Comment:** No further edits are needed in the current phase. All comments have been addressed properly by authors

**Response:** Dr. Mohamed F. Sallam, thank you for your previous revision, which greatly improved our manuscript. We are grateful to hear your positive feedback.

Reviewer 3:

**Comment:** The authors simulated the impacts of future temperature and CO_2_ conditions as well as predator presence on adult emergence and larval survivorship. They found no impact of the simulated climate conditions or predation risk on larval survivorship, but did find that adult emergence pattern was affected by the simulated climate conditions, with earlier, more uniform emergence under the intermediate climate conditions. This experiment was well-designed and the statistical analyses employed were appropriate. I would make sure that the discussion is clear in which SCCS had the statistically significant differences and why that might be and not conflating the impacts of predator presence/cues from this study and actual predation risk in a non-lab setting.

**Response:** Thank you for your comment. We answer your comments point-by-point below.

Overall comment

Abstract

**Comment:** Line 30: I would remove “those concerning” for clarity.

**Response:** Changed.

Introduction

**Comment:** Line 64: I would change to Around the world, some 390 million people are infected with dengue virus each year.

**Response:** Changed.

**Comment:** Line 108: Remove mainly

**Response:** Removed.

Methods

**Comment:** Line 215-219: This could be made a little clearer if you explicitly state what your response variable is for the linear mixed model. I think it is number of emergences per day, but the reader might not be 100% certain.

**Response:** Thank you for your observation. We add “affected the response variable, i.e. number of emergences” (lines 216-217).

**Comment:** Line 223-234: I think this is a really interesting way to analyze your data and you explained everything very thoroughly.

**Response:** Thank you for your observation and comment.

Results

**Comment:** Line 249: Could you specify how much higher the emergence rate was in the Light vs Control SCCS?

**Response:** We add this information in text, at lines 249-252.

“The first *Ae. aegypti* adult emergence was on day seven in the Extreme SCCS (n = 3 individuals), on day eight in the Intermediate SCCS (n = 11 individuals), while in the Light and Control SCCS the first adult emerged on day nine (n = 39 individuals; n = 3 individuals, respectively), with higher emergence rate in the Light SCCS.”

Discussion

**Comment:** Line 279-280: Since you only actually saw the accelerated development time under the intermediate climate change scenario, I would specify that here.

**Response:** We add this information in the first sentence in Discussion section “We showed that simulated climate change scenarios accelerate development time of *Ae. aegypti* larvae, which agrees with previous studies in western Amazonia [21], with a emergence peak on a single day in Intermediate SCCS.” (lines 282-284).

**Comment:** Line 285: Since your larvae weren’t under actual threat of predation (just subjected to chemical cues of predators). I don’t know that you have the grounds to broadly state that climatic variables are a stronger ecological driver than predation risk.

**Response:** We modified the sentence to make it clear that the effect of SCCS was stronger than predation risk, particularly those related to chemical and visual cues. Now it reads:

“We observed only SCCS did affect adult emergence pattern of *Ae. aegypti*, indicating that, in this study, climatic variables effects (temperature and CO_2_ concentration) are stronger ecological driver than predation risk, particularly those related to chemical and visual cues.” (lines 287-290).

**Comment:** Line 308: I would substitute predation risk with predation cues here.

**Response:** Changed.

**Comment:** Line 326-327: Should just say “emergence distribution of *Ae. aegypti*.” without including adults.

**Response:** “adults” were removed.

**Comment:** Line 328-329: Since you just saw the effects in the Intermediate treatment, I would be interested to see a discussion of others that have seen a similar non-effect in the Extreme scenario where conditions are too hot and no longer speeding up development.

**Response:** In the Extreme SCCS, adult emergence started before the other scenarios (see Figure 5). However, when we evaluate the emergence pattern (adult emergence per day for each replicate, captured by kurtosis and skewness) the Intermediate SCCS exhibit a peaked and right-distributed emergence pattern, while this pattern did not occur in the other three SCCS (adult emergence was more evenly distributed throughout the days). This does not mean that extreme conditions did not accelerate the development. We added a sentence in Discussion section including a reference regarding a nonlinear relationship between temperature and life history traits.

“Typically, the relationship between temperature and life history traits is non-linear [44], and species have a thermal optimum to complete their development. Temperatures above the thermal optimum decrease species performance (e.g. immature survival). Although emergencies started earlier in the Extreme SCCS, the Intermediate SCCS revealed a pronounced emergence on a single day (10^th^ day). (lines 334-338).

**Comment:** Line 338: Change to “It is important to carry out…”

**Response:** Changed.

**Comment:** Line 345: Same as previous comments, you can’t really say *Ae. aegypti* develops faster under SCCS—just the larvae develop faster under the Intermediate SCCS

**Response:** Thank you for your observation. We add “larvae” after *Ae. aegypti* in this sentence, we also reviewed this throughout the text.

Reviewer 4:

Minor edits

**Comment:** line 37: define ppmv

**Response:** We defined ppmv at line 37.

**Comment:** line 63 and throughout: “Zika” is always capitalized

**Response:** Thank you for your observation. “Zika” were corrected.

**Comment:** line 66: change “by” to “during”

**Response:** Changed.

Introduction

**Comment:** line 70: “In addition to… food webs”. This sentence is confusing; isn’t individual biomass an example of a direct effect of climate change on individuals?

**Response:** Thank you for your comment. Yes, the individual biomass is a direct effect of climate change on individuals, we moved this example to the previous line. Now it reads:

“Climate change affects biodiversity at multiple levels. It may cause shifts on biomass [9], metabolism and behavior [10] at the individual level and, at population level, it can alter species distribution via changes in local conditions. Consequently, community composition can be altered by climate change [11], changing ecosystems and food webs [12–14].” (lines 71-75).

**Comment:** lines 70-90: there is a lot of information in these paragraphs. Some of the information could be moved to the discussion section; overall the Introduction should be more concise. It may help to organize it with some numeric indications, for example “There are three ways in which predator-prey interactions are influenced by climate change….” This may improve the readability of this section.

**Response:** Thank you for your example, we used it in the second paragraph of the Introduction section. We also reorganize the ideas in this paragraph to make it more concise and improve the readability, according to your suggestion. Now it reads:

“Climate change affects biodiversity at multiple levels. It may cause shifts on biomass [9], metabolism and behavior [10], at the individual level and, at population level, it can alter species distribution via changes in local conditions. Consequently, community composition can be altered by climate change [11], changing ecosystems and food webs [12–14]. There are two ways in which predator-prey interactions are influenced by climate change. First, it can increase the metabolic rates of individuals, as a consequence of higher temperatures [12], affecting the ability of predators to forage, capture and handle prey. In this way, climate change may modify prey density (density-mediated interactions) [15]; Second, besides direct predation, climate change alter predator-prey interactions via production, transmission, and reception of chemical cues. Under such circumstances, both predator and prey may suffer reduction in their abilities to detect each other [16]. In predation risk situations, releases of chemical cues is common, and the detection of predator by prey through them can modify feeding behavior and/or development rates (trait-mediated interactions) [15].” (lines 71-84).

Methods

**Comment:** line 140: please include the definition for the abbreviation “ppmv”

**Response:** The definition of “ppmv” was included.

**Comment:** lines 223-233: can you include a brief explanation /example of how skewness/kurtosis represent emergence patterns. Something like: “A left-skewed emergence pattern would indicate the majority of the larvae emerging early on” and “A positive kurtosis would indicate most larvae emerging at the same time”. I think this would help the reader more immediately understand the inclusion of skewness/kurtosis metrics in an analysis of emergence.

**Response:** We add a sentence explained how skewness/kurtosis represent emergence patterns.

“For example, a right-skewed would indicate an early emergence of *Ae. aegypti*, whereas a left-skewed would indicate a late emergence.” (lines 225-227)… and “A positive kurtosis would indicate that most larvae emerge at the same time, whereas a negative kurtosis would indicate a more evenly emergence distribution.” (lines 231 -233).

Results

**Comment:** line 250: I think the phrase “emergence pattern” is confusing here – do you mean the day of first emergence? What exactly was being tested here. Generally, it seems that kurtosis and skewness are better representations of the overall emergence pattern. Can you clarify this section?

**Response:** We agree that the term “emergence pattern” is confusing here. We replace it to “emergence” only. Here, “emergence” means adult emergence per day for each replicate and not the day of first emergence. We agree that kurtosis and skewness are better representations of the overall emergence pattern, but we used a linear mixed model (LMM) (with Gaussian distribution) to detect if predation and SCCS and their interaction affect number of emergences, controlling for repeated measures and time autocorrelation. In this model we considered the replicates as a random factor (replicates were our repeated measure), with an autocorrelation function to control repetition across days, and SCCS and predation risk as fixed factors. This model is equivalent to a two-way ANOVA with repeated measures. The model output is not informative in terms of temporal patterning, because we did not include time as a fixed factor, so as to retain a viable number of degrees of freedom. To better understand this effect of time without including time in our models as fixed factor, we preferred to use the approach mentioned above (calculate kurtosis and skewness of distribution of emergence time).

Discussion

**Comment:** lines 287-288: do you mean “in some cases, an effect was perceived in the adult stage”

**Response:** Yes, you are right. We modify the sentence according to your suggestion, at lines 292-293.

**Comment:** line 327-329: The result that Intermediate had more of an effect than Extreme SCCS is not too surprising. There is some good literature to show that we would not expect a linear relationship between temperature and life traits. See Mordecai et al. “Thermal biology of mosquito‐borne disease” 2019. Ecology Letters https://doi.org/10.1111/ele.13335

**Response:** Thank you for your suggestion. We added a sentence in the Discussion section including the idea you gave us and citing this interesting paper. This information is at lines 334-337.

Overall Comments

**Comment:** the paper is nicely written; there are some areas where the writing could be more concise or more organized to improve readability (see specific comments above)

**Response:** Thank you for your comment. We reorganized the Introduction section according to your comment regarding lines 70-90.

**Comment:** another question of interest might be regarding direct predation instead of predation risk; an experiment could test the level of predation between these two species under the different climate scenarios by observing the survival of *Aedes aegypti* larvae with some predators (with the predators swimming freely outside of a cage). It seems that this would be a better reflection of real-life conditions. Was there a reason to examine predation risk specifically instead of measuring the effect of direct predation in these scenarios?

**Response:** Thank you for this comment. Indeed, this is an interesting idea to future experiments. We believe that our experiment is a good reflection of real-life conditions. In a natural environment (lakes, ponds, pools), *Ae. aegypti* larvae are exposed to both predation and predation risk, and prey behavior and density are affected by direct consumption of predator and by predator presence. *Ae. aegypti* larvae use different strategies to avoid predators, such as seeking shelter in macrophytes roots. In such habitats, they are under predation risk effects, which can lead prey to decrease the search for food resources and allocate energy in defense instead of development. As one of our focus in this study was to understand whether the predator stimulus (without direct consumption) could affect the *Ae. aegypti* emergence rates, if we used direct predation, the density of *Ae. aegypti* larvae could be greatly reduced or even completely consumed by the predator and we would not reach our goal. I

In a meta-analysis study, Preisser et al (2005) assessed the impact of direct predation (density mediated interaction, DMI), and predation risk (trait-mediated interactions, TMIs) on the interaction predator-prey. They showed that intimidation caused by non-lethal predation has high costs for prey, especially in aquatic environments, and is often overlooked.

We do find it important to include this information in the text “*Aedes aegypti* larvae use different strategies to avoid predators, such as seeking shelter in macrophytes roots. In such habitats, they are under predation risk effects, which can lead prey to decrease the search for food resources and allocate energy in defense instead of development.” (lines 151-155).

**Comment:** the use of skewness and kurtosis to represent the overall emergence pattern is interesting and clever. I think is a great way to summarize emergence patterns. Are there any examples in the literature of these metrics being used to analyze temporal patterns?

**Response:** Thank you for your comment. Yes, these metrics were used by other studies to analyze temporal patterns. For example:

- Siqueira et al. 2008. Species richness, abundance, and body size relationships from a neotropical chironomid assemblage: Looking for patterns. Basic and Applied Ecology. doi: https://doi.org/10.1016/j.baae.2007.06.002.

The authors evaluated temporal variation, using skewness, of body size of a chironomid assemblage.

- Falconi et al. 2020. Effects of data aggregation on time series analysis of seasonal infections. International Journal of Environmental Research and Public Health. doi: https://www.mdpi.com/1660-4601/17/16/5887

The authors used skewness and kurtosis to assess the daily, weekly, and monthly distribution of three respiratory and enteric infections. These data were used as a preliminary analysis to build a model for time series analysis that can be used for various diseases.
